# The Relational Playbook Nurse Leadership Development Program Using the Whistle Systems Employee Recognition Platform: Feasibility Mixed Methods Study

**DOI:** 10.2196/79188

**Published:** 2026-02-02

**Authors:** Marguerite Daus, Brigid Connelly, Drew Carter, Heather M Gilmartin

**Affiliations:** 1 Division of General Internal Medicine, Department of Medicine, University of Colorado Anschutz Medical Campus, 13001 E 17th Place, Aurora, CO, 80045, United States, +1 3037242261; 2 Denver/Seattle Center of Innovation for Veteran-Centered and Value Driven Care, United States Department of Veterans Affairs, VA Eastern Colorado Healthcare System, Aurora, CO, United States; 3 Whistle Systems, St. Louis, MO, United States; 4 Department of Health Systems, Management and Policy, School of Public Health, University of Colorado Anschutz Medical Campus, Aurora, CO, United States

**Keywords:** leadership, nursing, mobile app, development, workforce

## Abstract

**Background:**

Leadership development programs in health care often fail due to their lack of adaptability to the schedules of busy clinicians. This study addressed the need for scalable, flexible programs tailored to nurse leaders.

**Objective:**

This study evaluated the acceptability, appropriateness, and feasibility of the Relational Playbook, an evidence-based leadership development program developed in the Veterans Health Administration delivered through the Whistle Systems employee recognition web application and mobile app.

**Methods:**

A 1-year, single-team pilot was deployed using descriptive survey data and qualitative interview analysis. The Relational Playbook’s educational content and interventions were hosted on the Whistle platform, which integrates behavioral science and gamification strategies. Content was delivered weekly via app-based nudge notifications and email. Engagement metrics included activity completion rates. User experience data were collected through weekly reflection surveys (with Likert-scale responses and open-text options); monthly check-ins; and a postimplementation acceptability, appropriateness, and feasibility survey and interview. Descriptive statistics summarized engagement levels and trends, and qualitative data were analyzed using content analysis to identify recurring concepts. Quantitative and qualitative data were analyzed sequentially for comprehensive insights.

**Results:**

The section chief and 4 practicing cardiology nurse practitioners from a large academic medical center participated. The nurse practitioner section chief deemed the Whistle platform an acceptable, appropriate, and feasible technology for delivering the Relational Playbook content. They valued the weekly nudges, microlearning content, and flexibility of the web application and mobile app. The Relational Playbook content supported their personal growth and fostered positive shifts in attitudes toward work.

**Conclusions:**

Delivering leadership development content through the Whistle platform is an acceptable approach to support the growth and well-being of busy nurse leaders. The small sample and absence of a comparison group limit generalizability.

## Introduction

The well-being of nurse leaders and the nursing workforce is an urgent concern worsened by the COVID-19 pandemic, increasing patient complexity, evolving hospital systems, and high workloads [1]. Nurse leaders play a pivotal role in improving nurse well-being, patient care, and clinical outcomes by managing frontline clinical staff [2]. However, leadership is a challenging role that requires skills to foster interdisciplinary teamwork, continuous learning, and high reliability. Most nurse leadership training occurs through face-to-face didactic education or on-the-job training that falls short of true leadership development [3]. Digital leadership programs are available; however, most lack scientific rigor and impact evaluation [4]. With many nurse leaders nearing retirement, developing the next generation is essential to sustaining the profession and ensuring high-quality care [5].

The Relational Playbook is an innovative leadership development program grounded in adult learning principles, including experiential learning [6] and situated learning theories [7]. The Relational Playbook is designed to equip frontline clinical leaders with the skills to foster a culture of learning and high reliability within clinical teams. The Relational Playbook integrates evidence-based concepts and practices from positive psychology, team science, servant leadership, the Veterans Health Administration (VHA) Whole Health model, and clinical team training [8-10]. The Relational Playbook’s key innovation lies in bringing these principles together into a single, cohesive program tailored specifically for frontline health care leaders. These principles are presented in an e-book with five chapters on (1) creating a positive culture, (2) teamwork, (3) leading teams, (4) creating joy in work, and (5) communication and high reliability. The Relational Playbook contains brief asynchronous learning modules, 11 kick-off interventions, and 39 additional evidence-based interventions. Table 1 provides more details on the Relational Playbook chapters, their resource topics, and their kick-off interventions. Frontline leaders complete weekly self-directed education and then select and implement specific Relational Playbook interventions into existing meetings or trainings. Each chapter builds on the previous one and results in the development of supportive learning environments.

**Table 1.** Relational Playbook chapters, modules, and kick-off interventions.

Relational Playbook chapter	Resource topics	Kick-off interventions
Chapter 1: Creating a Positive Culture	Positive culture Assessing team well-being Appreciative inquiry	”Three Good Things” practice [11] Appreciative inquiry questions [12]
Chapter 2: Teamwork	Building a team Relationships at work Difficult relationships at work Hiring for high-performing teams	“Walk in My Shoes” exercise [13] Ice breaker questions [13]
Chapter 3: Leading Teams	Wellness-centered leadership Servant leadership Essential leadership skills	“Stop, Start, Continue” method [13] “Situation-Behavior-Impact” feedback [14]
Chapter 4: Creating Joy in Work	Burnout Joy and happiness Gratitude	Understanding what matters [15] “Was It Worth It?” method [16] Gratitude huddle [17]
Chapter 5: Communication and High Reliability	Effective communication High-reliability practices	Start-of-day huddles [18] Debriefs [18]

Pilot research with the Relational Playbook has suggested improvements in employee engagement and retention while reducing burnout and turnover, which are critical workforce challenges [8]. The Relational Playbook aligns with multiple priority areas in health care, including the shift toward learning health systems to improve patient safety. In 2022, the Relational Playbook was registered as an invention with the VHA Technology Transfer Program (VHA ID 2022-474) to foster partnerships with external digital technology innovators and leverage emerging technology to expand and scale the program.

The Relational Playbook team collaborated with Whistle Systems, a company specializing in digital programs and trainings to sustain employee behavior change and improve workplace culture. Leveraging a mobile-first design, the Whistle platform integrates evidence-based strategies such as microlearning, gamification, and strategic nudges to optimize user engagement and adherence [19]. The platform delivers real-time feedback through notifications, on-demand resources, and a user-centric interface to enhance accessibility. Whistle has shown measurable success in improving employee engagement and reducing turnover across sectors, including aviation, finance, and construction [20]. The partnership aimed to adapt Relational Playbook content to the Whistle platform and assess whether this innovative technology is an acceptable, appropriate, and feasible tool for delivering the Relational Playbook to nurse leaders.

## Methods

### Overview

We conducted a 1-year, single-team feasibility study of the Relational Playbook delivered on the Whistle platform within a real-world clinical setting using descriptive survey data and qualitative interview analysis. A cardiology nurse practitioner (NP) team (n=5) at a large academic medical center volunteered to pilot the Relational Playbook on Whistle driven by an interest in leadership development training to improve team dynamics. The NP section chief was the primary participant, and their team members were invited to engage with the platform to enhance team understanding and participation. To maintain participant confidentiality, detailed demographic information was not reported. The inclusion criterion was a formal supervisory role in the department. Participation was voluntary and considered part of the employees’ work.

The Relational Playbook and Whistle team established a cooperative research and development agreement enabling collaboration between the VHA and private companies. The first author adapted the Relational Playbook content for delivery on the Whistle platform with input from the Relational Playbook developers (HMG and BC) and Whistle engineers. The educational content was reformatted into a microlearning flash card model incorporating short text, colored images, videos, and kick-off interventions (Multimedia Appendix 1). The Whistle-enabled Relational Playbook used multiple behavioral science mechanisms, including celebratory feedback (confetti) to trigger dopaminergic reward responses, progress indicators (completion bars) to leverage the goal gradient effect, microlearning modules to reduce cognitive load and enhance perceived task simplicity, and strategic nudges to serve as action triggers and mitigate decision inertia. The Whistle web platform hosted the content, with accessible iPhone and Android mobile apps.

**Figure 1.**
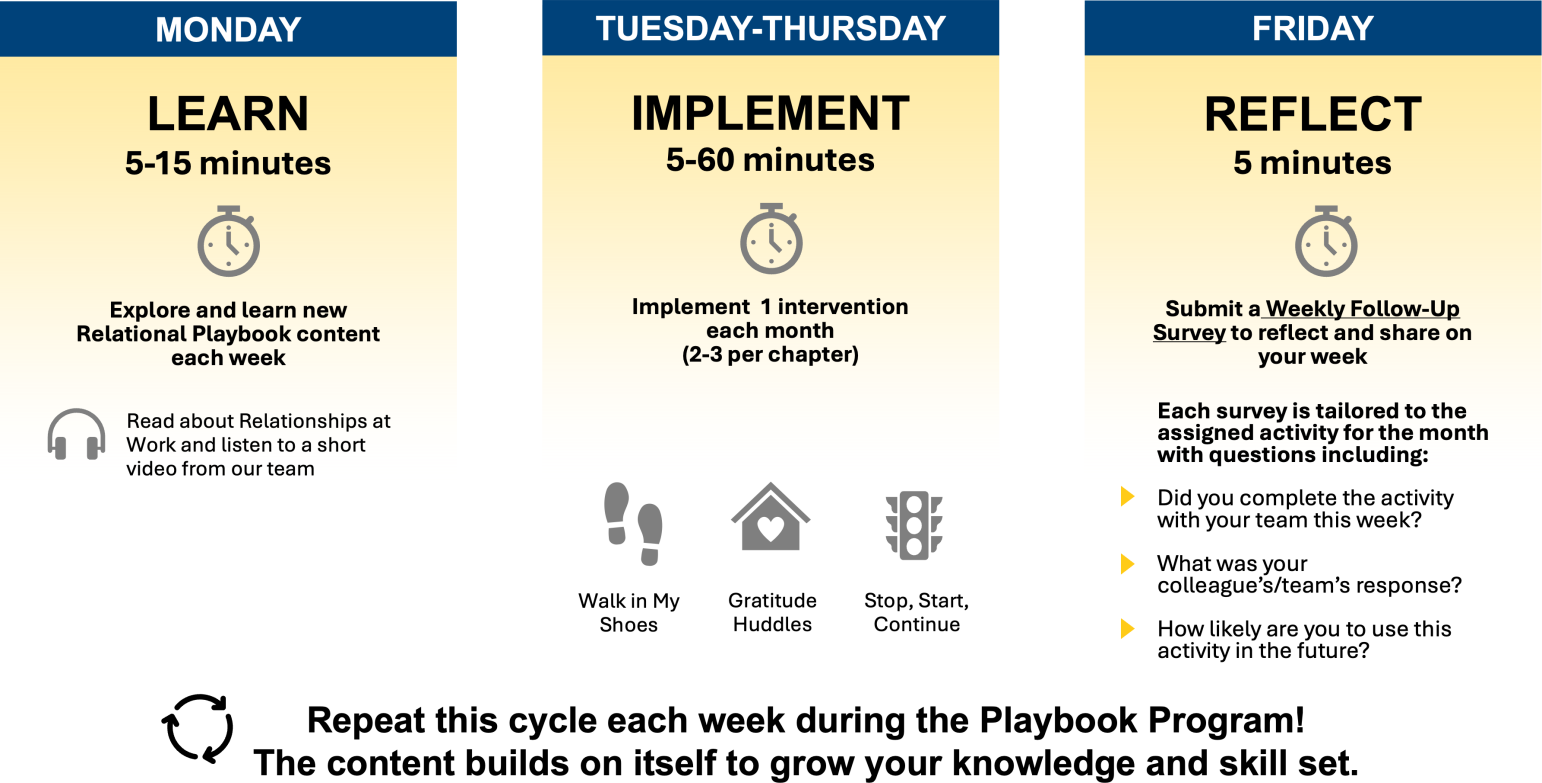
The Relational Playbook program weekly activities. This figure shows what a week in the Relational Playbook looks like for a cardiology nurse practitioner team. On Monday, they are instructed to take 5 to 15 minutes and learn new content by completing learning modules and watching short videos. The rest of the week, they implement their learnings and activities through 1 intervention each week, such as “Walk in My Shoes” or “gratitude huddles.” At the end of the week, they submit a weekly follow-up survey that is tailored to the assigned activity for the month to see whether they progressed with the activity and their team’s response.

The Relational Playbook on Whistle begins with participants completing the 13-item Learning Environment Assessment Tool (Multimedia Appendix 2), an abbreviated version of the validated 64-item Learning Environment Survey [10,21]. The Learning Environment Assessment Tool evaluates key aspects of supportive learning environments through statements such as “The cardiology team demonstrates trust and mutual respect with each other,” “The cardiology team is comfortable asking for help and feedback from others,” and “The cardiology team can control their own practice and regularly participate in decisions about their work.” Each item is rated on a 3-point scale (1=“rarely,” 2=“sometimes,” and 3=“almost always”). Responses are automatically summed in Qualtrics (Qualtrics International Inc) and ranked from lowest to highest. Using these data, the research team assigned initial (lowest ranking) and subsequent chapters to the participants in order. The Relational Playbook consists of 5 chapters, each delivered over a 2-month interval across 1 year (Figure 1). Participants receive weekly email and app notifications (“nudges”) linking to flash card–based learning modules. Each module concludes with a brief comprehensive quiz and celebratory feedback to reinforce engagement. Modules end with details about the next chapter’s kick-off intervention to implement.

The evaluation data were collected through surveys and interviews from the NP section chief (eg, primary participant) to measure implementation, engagement, and adoption outcomes. Primary implementation outcomes were the acceptability, appropriateness, and feasibility of the Relational Playbook on Whistle assessed using the Acceptability of Intervention Measure, Feasibility of Intervention Measure, and Intervention Appropriateness Measure [22] (Multimedia Appendix 3). These surveys assess the acceptability, appropriateness, and feasibility of the Relational Playbook on Whistle through statements such as “The Relational Playbook on Whistle meets my approval,” “The Relational Playbook on Whistle seems suitable,” and “The Relational Playbook on Whistle seems implementable.” Each item is rated on a 5-point scale (1=“completely disagree”; 5=“completely agree”) and analyzed using mean scores [22]. The secondary outcomes of engagement and adoption were assessed using all participant platform visit data and survey responses (n=5), supplemented by a follow-up interview with the NP section chief to contextualize the findings. Whistle’s reporting tools were used to track platform visits, module completions, survey completions, and responses.

Quantitative data, including platform use and survey responses, were summarized both by chapter and for the Relational Playbook overall. Chapter and Playbook completion rates were calculated as the percentage of completed modules relative to the total number of modules using Microsoft Excel. Platform visit data were reviewed to confirm that all participants accessed the Relational Playbook content on Whistle at least once. For qualitative data, we used a rapid qualitative matrix approach [21]. Two team members (BC and MD) met to discuss data and reach consensus on the concepts; given the informal nature and small sample (n=1), all data were analyzed instead of stopping at a point of saturation. The initial matrix summary was developed by 1 team member using identified concepts and illustrative quotes from the data. A second team member independently reviewed and refined the matrix to ensure accuracy, completeness, and consistency in data representation. To enhance rigor, discrepancies were discussed and resolved collaboratively. The full analytic team then conducted a review of the finalized matrix, engaging in consensus building to identify cross-cutting concepts and key insights.

### Ethical Considerations

This study was deemed an exempt human research study by the Colorado Multiple Institutional Review Board (17-1153) and did not require informed consent. All participant data were handled in accordance with institutional privacy and confidentiality guidelines.

## Results

The NP section chief and 4 cardiology NP team members participated in the study, with the NP section chief acting as the primary implementer and evaluator due to their formal supervisory role. The NP team provides clinical care across various inpatient and outpatient settings (eg, heart failure clinic and structural heart and valve clinic) and meets virtually each month for updates and professional development.

### Acceptability, Appropriateness, and Feasibility and Platform Visits

**Figure 2.**
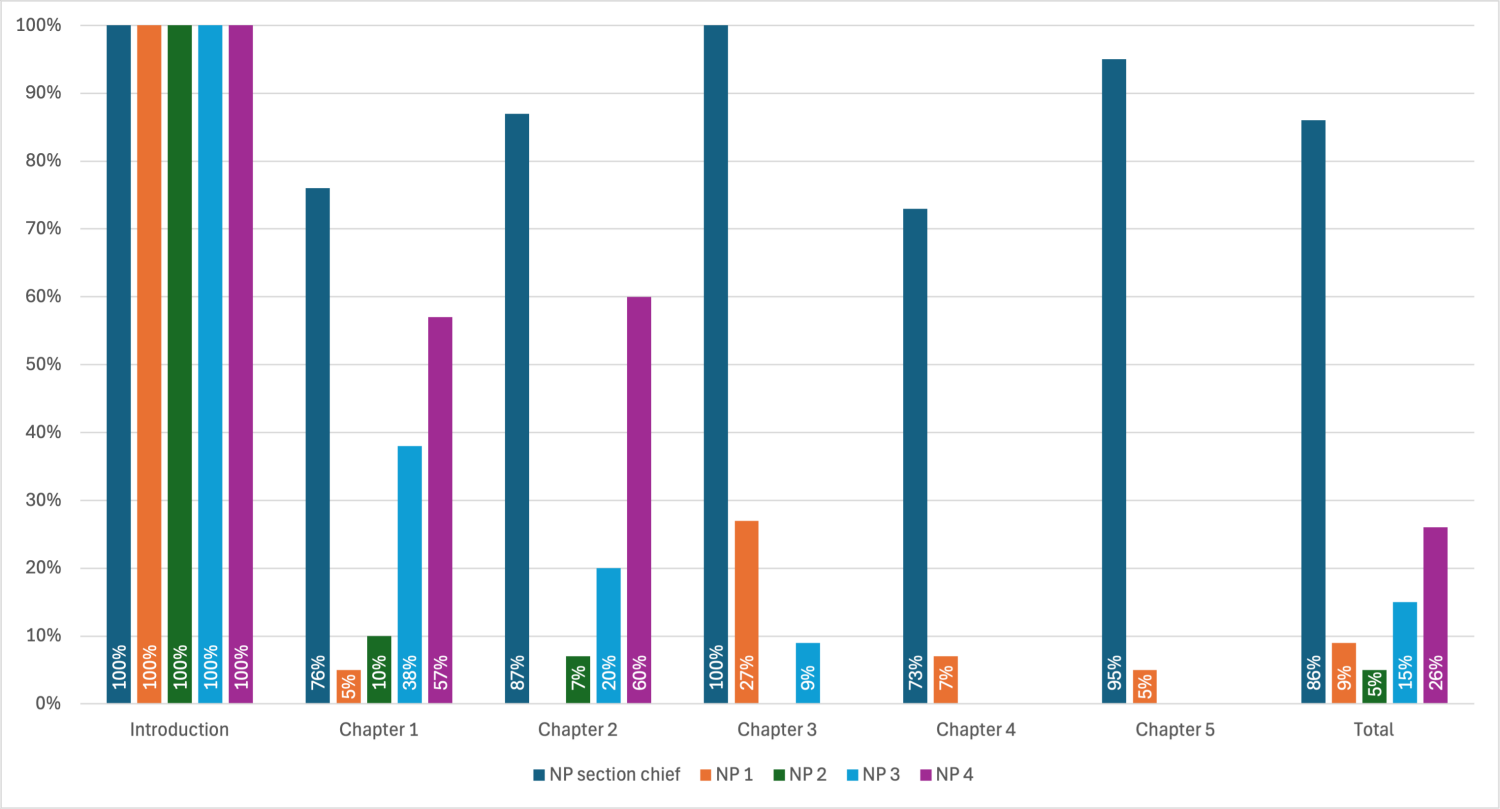
The Relational Playbook on Whistle completion rates. This figure displays the chapter completion rates for each participant alongside their total completion rate for the entire program. While all participants completed the introduction chapter, participation rates declined for everyone but the nurse practitioner (NP) section chief for chapters 1 to 5.

The NP section chief, the primary participant, strongly agreed (5/5) on all measures that the Relational Playbook on the Whistle platform was acceptable (Acceptability of Intervention Measure), appropriate (Intervention Appropriateness Measure), and feasible (Feasibility of Intervention Measure). They engaged with 86% (73/85) of the learning content and reflection surveys and adopted all 11 kick-off interventions at least once with their team. The 4 cardiology NP team members engaged fully with the introduction, and their engagement declined over the subsequent 5 chapters. During scheduled check-ins, NP team members reported that their busy schedules, their direct care responsibilities, and not leading their own teams made it harder for them to implement the Relational Playbook interventions as they were not directly applicable to their roles. They found the learning components valuable and noted that these resources enhanced their understanding of and engagement in the section chief’s activities. However, the lack of applicability in their daily work led to disengagement with the platform. Figure 2 shows the completion rates by chapter for the Whistle-enabled Relational Playbook.

The NP section chief provided generally positive feedback on the kick-off interventions and reported that their team adopted a more positive outlook, identifying what mattered most to them, such as “‘family,’ ‘friends/relationships,’ ‘fulfilling my responsibilities,’ and ‘being good at my job.’” They had specific successes with using debriefs:

Worked well with a co-worker to solve a problem!

However, the *Walk in My Shoes* and *Was It Worth It* interventions were more challenging to implement, as the NP section chief explained:

...I think it needs to be the right timing and people.

They also reported adapting some interventions, such as replacing the “Stop, Start, Continue” group discussion with an online survey and incorporating the “What was a win this week” question from chapter 4 as a meeting icebreaker.

### Interview Results

#### Platform Usability and Learning Content

The NP section chief primarily used the website, rating it as easy to use (4 out of 5 on a verbal ease-of-use scale) and preferable to the mobile app, which required frequent log-ins and multiple clicks to access content. They appreciated the ability to navigate seamlessly through the program and liked the push notifications (ie, nudges). The Relational Playbook content was described as “bite size enough to be done in one sitting and...easily digested.” However, they observed that the Relational Playbook was designed for teams that “work together in a clinical way.” Considering that the NP group did not physically work together, they suggested modifying the content to better reflect virtual team dynamics.

#### Relational Playbook Implementation

The NP section chief rated the implementation of the Relational Playbook practices as moderately easy (3 out of 5 on a verbal ease-of-implementation scale). They confirmed that the practices fostered a more positive team outlook, stating the following:

...there’s some hard days.... And we focus a lot on the negative...instead of what went right. I liked that about the Playbook.

They emphasized that the learning content only took 5 to 10 minutes to complete. Overall, the NP section chief described the Relational Playbook on Whistle as a valuable program:

You can always improve as a leader—I highly recommend it.

#### Adaptations and Sustainment

The NP section chief suggested adaptations before expanding the program: (1) tailor Relational Playbook content for virtual teams that do not meet routinely in person, (2) condense the program to 6 months (from 1 year), (3) reduce nudges to every other week (from weekly), and (4) develop an educational module for team members without formal leadership roles. At the conclusion of the 1-year program, the NP section chief reported ongoing use of the huddles and appreciative inquiry practices.

## Discussion

This feasibility study demonstrated that the Relational Playbook on Whistle is an acceptable, appropriate, and feasible intervention for a nurse leader. The NP section chief engaged with 86% (73/85) of the educational content and implemented all 11 kick-off interventions, confirming the platform’s usability. Participant feedback emphasized the Relational Playbook’s strengths, including its concise, “bite-sized” content; intuitive navigation; and direct relevance to clinical practice. By applying Relational Playbook practices, the NP section chief fostered a culture of learning and positivity within the team. The NP clinical team accessed the platform and participated intermittently. This may reflect the absence of opportunity for practicing NPs to put the leadership interventions into practice, the unique challenges of virtual teams that do not routinely work in person (noted above), or the assumption that leadership development is only for those who have formal leadership titles. Their reports of finding the education components valuable reinforce the section chief’s recommendation for an educational module for team members without formal leadership roles.

The Whistle platform behavioral science features guided participants to set learning intentions, assume responsibility for their goals, and receive feedback on progress. While our study data did not allow for a granular analysis of each feature’s individual contribution to engagement, this is an area that we will explore in future research. Rapid application of newly acquired skills in practice represents the gold standard of leadership development programs. Our single-team study process and findings align with recent work by Güntner et al [23], which reported that a web-based leadership transfer intervention positively influenced leaders’ mindsets and self-regulated learning. The significance of these studies lies in demonstrating that digital microlearning interventions can effectively support leadership development in high-demand clinical environments, offering a scalable and cost-efficient alternative to traditional programs. Future studies of the Relational Playbook on Whistle will advance the evidence base for digital leadership training programs, ensuring positive outcomes in a cost-effective manner.

The partnership with Whistle Systems was an opportunity to integrate an evidence-based leadership development program into a digital technology innovation that delivered microlearning content using behavioral science and gamification principles. This technology overcame time constraints, a common barrier for busy nurse leaders [24,25], and promoted engagement and new habits. The Whistle platform’s usability was rated favorably by the NP section chief, with the website preferred over the mobile app due to frequent re–log-ins and navigation challenges. Nudges effectively promoted engagement by linking directly to assigned content; however, a decrease in frequency was suggested to reduce response burden.

Additional behavior-driving tools offered by the Whistle platform were not leveraged in this feasibility study that could increase engagement and effectiveness among team members. These include real-time automated acknowledgments; hospital-branded Visa cards for monetary reward; peer or community recognition engagement through Whistle’s “Town-Square” social feature; or the platform’s artificial intelligence engine Robin, which tailors nudges informed by behavioral personalization algorithms to effectively prompt individuals while considering their unique motivational drivers [19]. Future iterations of the Relational Playbook on Whistle will include an educational module for team members in clinical roles to motivate them to learn and engage in culture change, content for virtual and hybrid teams, and additional Whistle tools.

This study’s strengths include the real-world evaluation of the Relational Playbook on Whistle and its focus on clinical leadership development. The feasibility approach provided early insights into implementation outcomes, user experience, and potential program impact. However, several limitations should be noted: the small sample size and single-team design, reliance on 1 primary participant, absence of a control group, and lack of objective leadership or patient-related outcomes reduce the ability to attribute observed changes to the intervention. These factors limit generalizability, and findings should be interpreted with caution. Future research will address these limitations by including a larger, more diverse sample; detailed demographic data; a comparison group; and multisource ratings to capture changes in leader behavior and team culture before and after implementation.

The Relational Playbook on Whistle shows strong promise as an acceptable, appropriate, and feasible nurse leadership development program capable of addressing critical workforce challenges such as burnout, team dynamics, and leadership readiness. To maximize its impact, future iterations should include adaptations for virtual teams, streamline program delivery, actively engage all team members, assess the impact on patient safety, and evaluate cost-effectiveness compared to traditional leadership programs. Scaling the Relational Playbook across diverse clinical settings will require strategic collaboration with technology partners to ensure accessibility, flexibility, and sustained implementation. By leveraging digital platforms for leadership development, health care organizations can accelerate skill acquisition, strengthen team culture, and build resilient leaders in an increasingly complex care environment.

## Supplementary material

10.2196/79188 Multimedia Appendix 1 Screenshots from the Whistle platform showing its unique features, with the Relational Playbook's educational content reformatted into a microlearning flash card model incorporating short text, colored images, videos, and kick-off interventions.

10.2196/79188 Multimedia Appendix 2 The 13-item Learning Environment Assessment Tool (LEAT) is an abbreviated version of the validated 64-item Learning Environment Survey. The LEAT assesses key aspects of supportive learning environments, and each item is rated on a 3-point scale (1=“rarely,” 2=“sometimes,” and 3=“almost always”).

10.2196/79188 Multimedia Appendix 3 Primary implementation outcomes assessed using the Acceptability of Intervention Measure, Feasibility of Intervention Measure, and Intervention Appropriateness Measure tailored to the Relational Playbook on Whistle. Each item is rated on a 5-point scale (1=“completely disagree”; 5=“completely agree”) and analyzed using mean scores.

## Abbreviations

NP: nurse practitioner
VHA: Veterans Health Administration
