# Supplementary material for: The Relational Playbook Nurse Leadership Development Program Using the Whistle Systems Employee Recognition Platform: Feasibility Mixed Methods Study
Source: JMIR Nurs. 2026 Feb 2;9:e79188. doi: 10.2196/79188 (PMC12863652; doi:10.2196/79188)
Supplement: Multimedia Appendix 1 [file nursing-v9-e79188-s001.docx]

**Appendix 1: Whistle Platform Screenshots Showcasing Unique Features**

**
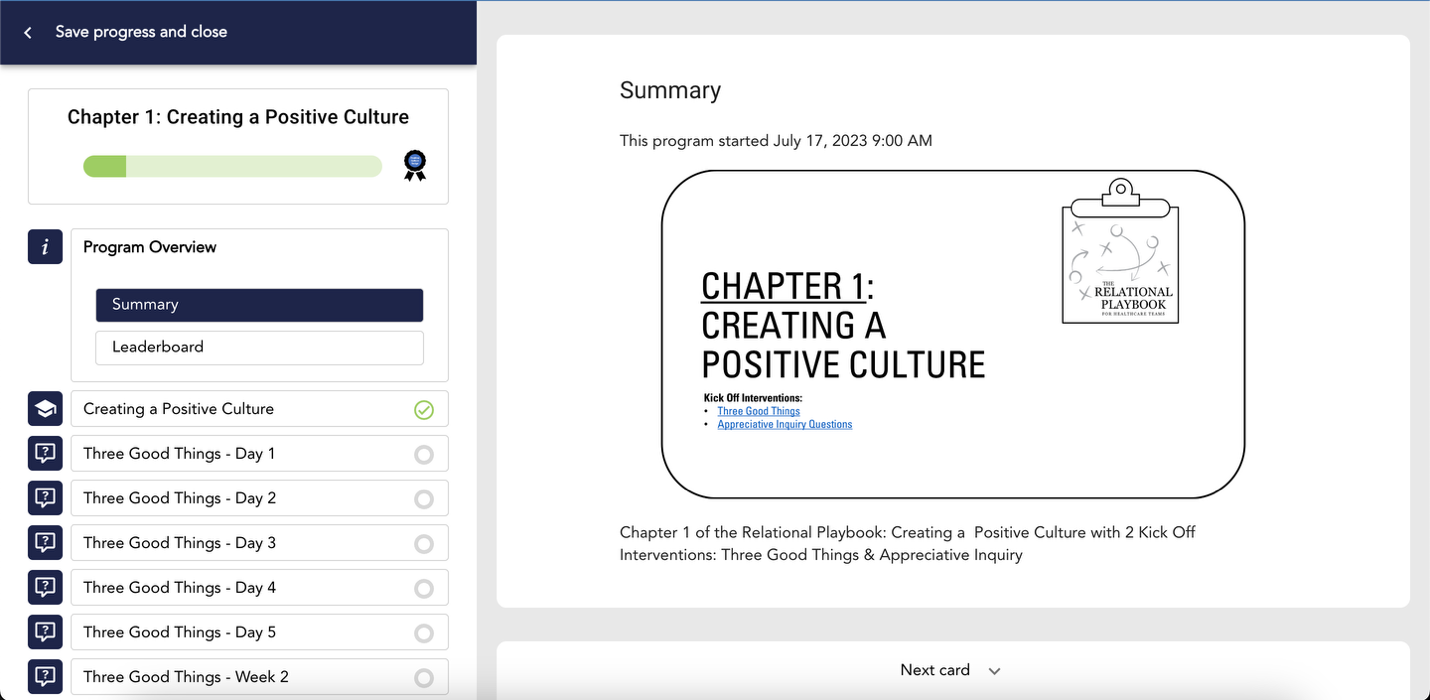
**

**
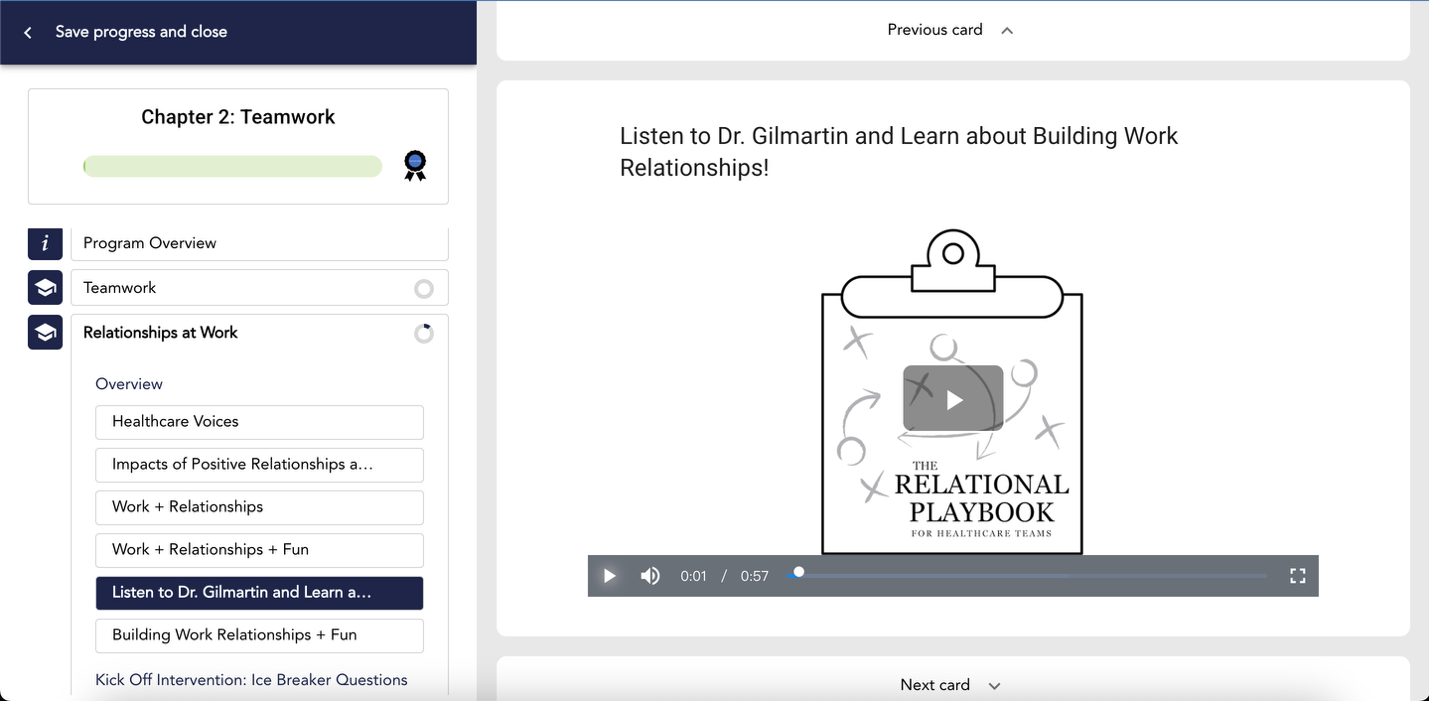
**

**
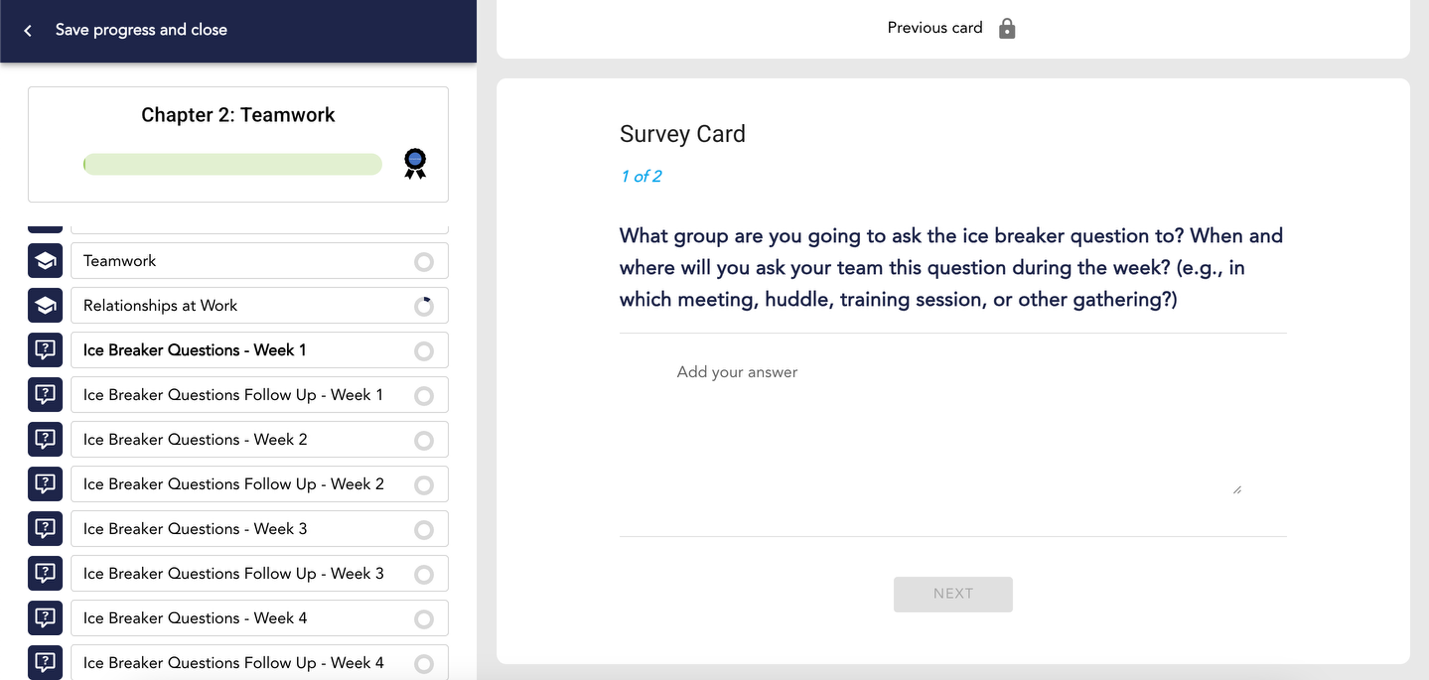
**


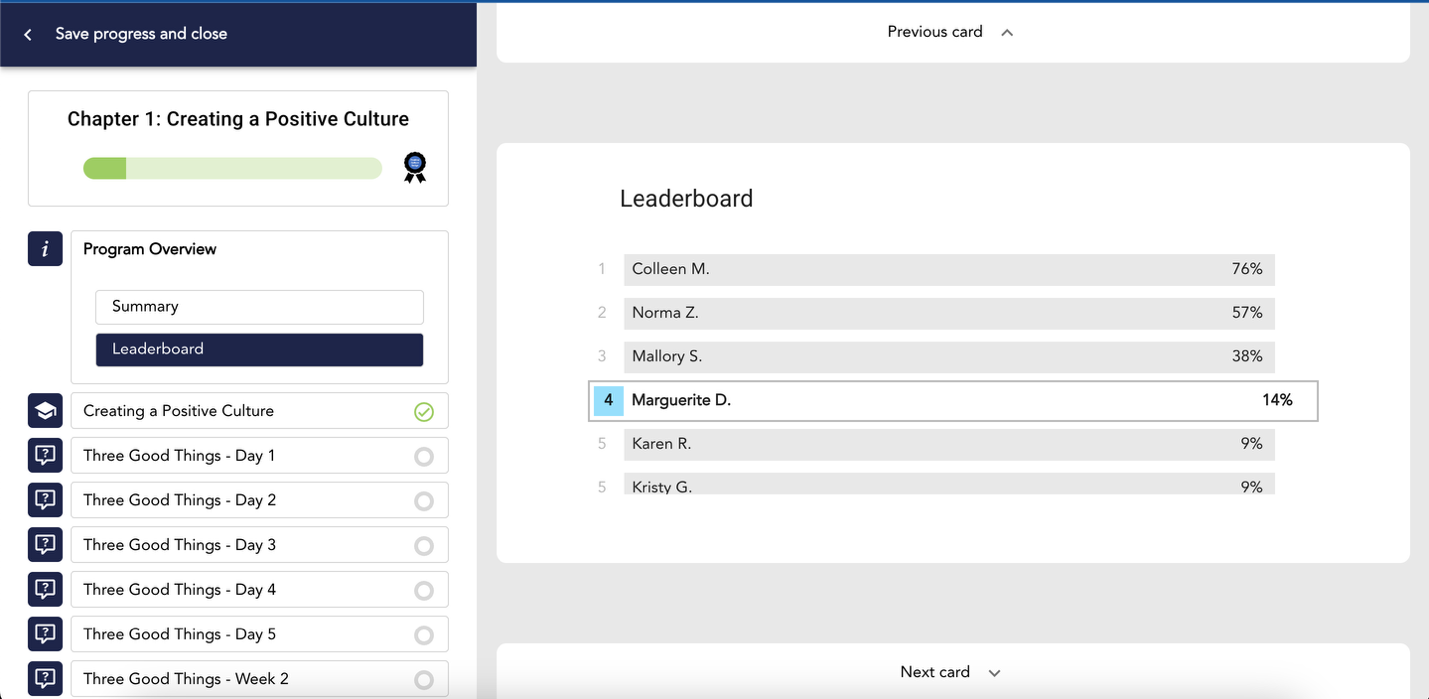


Participant 1

Participant 2

Participant 3

Participant 4

Participant 5

Participant 6
