# Supplementary material for: The Relational Playbook Nurse Leadership Development Program Using the Whistle Systems Employee Recognition Platform: Feasibility Mixed Methods Study
Source: JMIR Nurs. 2026 Feb 2;9:e79188. doi: 10.2196/79188 (PMC12863652; doi:10.2196/79188)
Supplement: Multimedia Appendix 2 [file nursing-v9-e79188-s002.docx]

**Appendix 2: 13-item Learning Environment Assessment Tool**

High-quality relationships, communication and a culture that supports learning are the backbone of high-performing clinical teams. Do you know how your team is doing?

The Learning Environment Assessment Tool will help you assess the state of relationships, communication and learning within your team or department. The aim of this work is to help move teams from good to great while improving employee engagement and safety climate and lowering burnout and turnover.

Instructions: Rate your team on a scale of rarely (1 point), sometimes (2 points), or almost always (3 points) for the following statements

1. The cardiology team demonstrates trust and mutual respect with each other.

2. The cardiology team has a good understanding of each other’s talents and skills.

3. The cardiology team recognizes and values humor, joy, and celebration in the workplace.

4. Cardiology leaders and staff are comfortable having difficult conversations.

5. The cardiology team talks about their mistakes and ways to learn from them.

6. The cardiology team is comfortable asking for help and feedback from others.

7. Cardiology leaders and staff exhibit active listening skills, curiosity and are willing to take part in team building activities.

8. The cardiology team can control their own practice and regularly participate in decisions about their work.

9. The cardiology team regularly uses huddles or debriefs to identify problems and reflect on past performance.

10. The cardiology team trains as a team and conducts dry runs to improve performance.

11. Cardiology leaders hire new employees based on their willingness to learn, work, and communicate well with others

12. The cardiology team uses highly reliable communication skills.

13. The cardiology team discusses their well-being and levels of stress and burnout.

Thank you for completing the Learning Environment Assessment Tool.

Your responses are described below and have been grouped into topics covered by The Relational Playbook. These topics have been shown to improve relationships and communication in teams:

***Here's how you scored in each category:***

**Creating a Positive Culture: (X/Score) [out of 15 points]**

If you scored...

13-15: This is an area of strength

5-12: This is an area for improvement

**Teamwork: (X/Score) [out of 24 points]**

If you scored...

21-24: This is an area of strength

8-20: This is an area for improvement

**Leading a Team: (X/Score) [out of 9 points]**

If you scored...

8-9: This is an area of strength

3-7: This is an area for improvement

**Creating Joy in Work: (X/Score) [out of 6 points]**

If you scored...

5-6: This is an area of strength

2-4: This is an area for improvement

**Communication and High Reliability: (X/Score) [out of 15]**

If you scored...

13-15: This is an area of strength

5-12: This is an area for improvement

For more information about how to interpret your Learning Environment Assessment Tool responses, return to the Relational Playbook.
