# Supplementary material for: The Relational Playbook Nurse Leadership Development Program Using the Whistle Systems Employee Recognition Platform: Feasibility Mixed Methods Study
Source: JMIR Nurs. 2026 Feb 2;9:e79188. doi: 10.2196/79188 (PMC12863652; doi:10.2196/79188)
Supplement: Multimedia Appendix 3 [file nursing-v9-e79188-s003.docx]

Appendix 3: Acceptability, Appropriateness and Feasibility Measures

The Acceptability, Appropriateness and Feasibility Survey measures by Weiner et al, (2017) are associated with adoption of evidence-based practices The measures are defined as:

- **Acceptability** = The perception among stakeholders that the [Playbook on Whistle] is agreeable or satisfactory
- **Appropriateness** = The perceived fit, relevance, or compatibility of the [Playbook on Whistle] for [Cardiology Teams] and to address [e.g., the lack of relationship and communication education and practices for healthcare teams]
- **Feasibility** = The extent to which the [Playbook on Whistle] can be successfully used or carried out with [Cardiology Teams]

Weiner, B. J., Lewis, C. C., Stanick, C., Powell, B. J., Dorsey, C. N., Clary, A. S., ... & Halko, H. (2017). Psychometric assessment of three newly developed implementation outcome measures. *Implementation Science*, *12*(1), 1-12.

**Acceptability Measure**

|  | Completely disagree | Disagree | Neither agree nor disagree | Agree | Completely agree |
| --- | --- | --- | --- | --- | --- |
| 1. The [Playbook on Whistle] meets my approval. | ➀ | ➁ | ➂ | ➃ | ➄ |
| 2. The [Playbook on Whistle] is appealing to me. | ➀ | ➁ | ➂ | ➃ | ➄ |
| 3. I like the [Playbook on Whistle]. | ➀ | ➁ | ➂ | ➃ | ➄ |
| 4. I welcome the [Playbook on Whistle]. | ➀ | ➁ | ➂ | ➃ | ➄ |

**Appropriateness Measure**

|  | Completely disagree | Disagree | Neither agree nor disagree | Agree | Completely agree |
| --- | --- | --- | --- | --- | --- |
| 1. The [Playbook on Whistle] seems fitting. | ➀ | ➁ | ➂ | ➃ | ➄ |
| 2. The [Playbook on Whistle] seems suitable. | ➀ | ➁ | ➂ | ➃ | ➄ |
| 3. The [Playbook on Whistle] seems applicable. | ➀ | ➁ | ➂ | ➃ | ➄ |
| 4. The [Playbook on Whistle] seems like a good match. | ➀ | ➁ | ➂ | ➃ | ➄ |

**Feasibility Measure**

|  | Completely disagree | Disagree | Neither agree nor disagree | Agree | Completely agree |
| --- | --- | --- | --- | --- | --- |
| 1. The [Playbook on Whistle] seems implementable. | ➀ | ➁ | ➂ | ➃ | ➄ |
| 2. The [Playbook on Whistle] seems possible. | ➀ | ➁ | ➂ | ➃ | ➄ |
| 3. The [Playbook on Whistle] seems doable. | ➀ | ➁ | ➂ | ➃ | ➄ |
| 4. The [Playbook on Whistle] seems easy to use. | ➀ | ➁ | ➂ | ➃ | ➄ |
